# An educational approach based on a non-injury model compared with individual symptom-based physical training in chronic LBP. A pragmatic, randomised trial with a one-year follow-up

**DOI:** 10.1186/1471-2474-11-212

**Published:** 2010-09-17

**Authors:** Pia H Sorensen, Tom Bendix, Claus Manniche, Lars Korsholm, Dorte Lemvigh, Aage Indahl

**Affiliations:** 1 The Back Research Center, part of Clinical Locomotion Science, Funen Hospital, Ringe, and the University of Southern Denmark, Denmark; 2 Actual Address: Research Center for Back Diseases, Glostrup University Hospital, H29, Ndr. Ringvej 57, DK-2600 Glostrup, Denmark; 3 Department of Statistics, University of Southern Denmark, Odense, Denmark; 4 Department of Physical Medicine and Rehabilitation, Kysthospitalet Stavern, Norway

## Abstract

**Background:**

In the treatment of chronic back pain, cognitive methods are attracting increased attention due to evidence of effectiveness similar to that of traditional therapies. The purpose of this study was to compare the effectiveness of performing a cognitive intervention based on a non-injury model with that of a symptom-based physical training method on the outcomes of low back pain (LBP), activity limitation, LBP attitudes (fear-avoidance beliefs and back beliefs), physical activity levels, sick leave, and quality of life, in chronic LBP patients.

**Methods:**

The study was a pragmatic, single-blind, randomised, parallel-group trial. Patients with chronic/recurrent LBP were randomised to one of the following treatments: 1. *Educational programme *: the emphasis was on creating confidence that the back is strong, that loads normally do not cause any damage despite occasional temporary pain, that reducing the focus on the pain might facilitate more natural and less painful movements, and that it is beneficial to stay physically active. 2. *Individual symptom-based physical training programme *: directional-preference exercises for those centralising their pain with repetitive movements; 'stabilising exercises' for those deemed 'unstable' based on specific tests; or intensive dynamic exercises for the remaining patients. Follow-up questionnaires (examiner-blinded) were completed at 2, 6 and 12 months. The main statistical test was an ANCOVA adjusted for baseline values.

**Results:**

A total of 207 patients participated with the median age of 39 years (IQR 33-47); 52% were female, 105 were randomised to the educational programme and 102 to the physical training programme. The two groups were comparable at baseline. For the primary outcome measures, there was a non-significant trend towards activity limitation being reduced mostly in the educational programme group, although of doubtful clinical relevance. Regarding secondary outcomes, improvement in fear-avoidance beliefs was also better in the educational programme group. All other variables were about equally influenced by the two treatments. The median number of treatment sessions was 3 for the educational programme group and 6 for the physical training programme group.

**Conclusions:**

An educational approach to treatment for chronic LBP resulted in at least as good outcomes as a symptom-based physical training method, despite fewer treatment sessions.

**Trial registration:**

Clinicaltrials.gov: # NCT00410319

## Background

Increased attention is being directed towards cognitive behavioural issues in the management of chronic low back pain (cLBP). One reason is that cognitive interventions have generally demonstrated effectiveness in self-reported disability[1-5] and sick leave[5-12] similar to that of traditional treatments. Another reason is that benefits from traditional physical treatments of cLBP are generally only of moderate clinical efficacy[13].

A cognitive approach in this context is not a well-defined term. It covers a spectrum from an educational approach to cognitive behavioural treatments delivered by psychologists. For LBP, our impression is that most reported cognitive interventions can be classified as educational approaches designed to produce altered behaviour through insight and understanding, and not psychological treatments as such. A crucial part of such interventions will be what kind of insight emerges, how effectively it is delivered, and the person's internalisation of the message received.

Cognitive theories include elements of central pain perception as well as optimisation of peripheral muscular control[14-23].

Among several cognitive/educational models, the one by Indahl has been shown to be particularly effective regarding return-to-work[6,8,9]. This was so in the early 1990s, where the alternative was often a "be-careful-with-your-back!" attitude. His brief intervention (about 3 hours) was based on a 'non-injury model' intended to focus on the back as a strong structure, where pain was not to be taken as a sign of injury caused by any wrongdoing or 'inappropriate' behaviour, and that natural movements are more appropriate than movements influenced by uncertainty and a focus on carefulness [15]. The robustness of the spine seems well elucidated: It can withstand most kinds of 'abuse' even over years [24-28]. In some studies, loading even seems to protect the intervertebral discs,[29] indicating that discs respond to physical loading as do most other connective tissues [30,31].

In contrast, the 'injury model,' where pain is taken as a sign of injury from increased loading, seems not to have been useful as a basis for clinical management[32,33]. In common LBP it has not been possible so far to substantiate the nature of injury or spinal structure affected. This has left us with uncertainty as to how to manage much cLBP, reflected in the wide range of treatments recommended from bed rest to functional restoration. The injury model has unfortunately been useful in medicalising common LBP, but useless clinically to answer even simple questions from patients such as: What is wrong? Why do I hurt? When is the spine strong enough for activity? For other musculoskeletal injuries such as broken legs or sprained ankles, answers are available, but this is not the case for common LBP.

It is well known that certain jobs and working positions are associated with increased back pain[34,35]. However, it is not clear how far daily physical loading causes LBP or whether having an existing pain leads to greater problems in physically demanding jobs. Regardless of which is the cause and which is the effect, the injury model does not seem to be adequate in explaining degeneration or common painful conditions.

Regarding exercises and other physical therapies in cLBP, randomised controlled trials (RCT) generally show small effect sizes on a group basis. However, subgroup analyses suggest greater effectiveness for such treatments in people with particular clinical profiles. Individualised, symptom-based physical training programmes had, at the preparation of our study, shown success in an RCT with acute patients[36] and with sciatica patients[37]. Moreover, for those whose pain centralised with McKenzie assessment procedures, benefits were documented for directionally-preferred exercises[38]. Furthermore, the benefit of stabilising exercises had been demonstrated in one large[39] and two smaller studies[40,41]. Also for such exercises, it seemed that for particular subgroups deemed 'unstable', stabilising exercises were effective. Intensive exercises had previously also shown some efficacy[13].

The specific aims of this study were to compare the effects of prescribing for cLBP patients either:

- an educational approach designed to improve confidence in the robustness of the spine, or

- symptom-based physical training treatment

on the primary outcomes of back pain and activity limitation, and secondary outcomes of LBP attitudes (fear-avoidance/back beliefs), physical activity levels, work ability, quality of life, sick leave, and a number of other health-care treatments.

## Methods

### Study Design

The study was a pragmatic, single-blinded, randomised, parallel-group trial with follow-up periods of 2, 6 and 12 months. It was conducted in accordance with The Declaration of Helsinki 2000 and approved by the local Research Ethics Committee (ref. no. VF 20040016).

### Study Population

CLBP patients were recruited from the clinic at a multidisciplinary non-surgical Back Center. The patients were referred to the clinic from general practitioners and chiropractors from across the Funen county in Denmark. Most had already had various treatments, with less than satisfactory outcomes.

The following inclusion criteria were chosen: 18 to 60 years of age, LBP for at least 4 out of the previous 12 months and a mean LBP score over the last 14 days of ≥ 4 (scale 0-10). The back pain had to be greater than any associated leg pain. Exclusion criteria included the presence of cancer, traditional inflammatory diseases (Bechterew, Reiters disease, etc.), sequelae after earlier back surgery, conditions of competing joint or muscle disease, psychiatric illnesses, or any general disease that would hinder intensive physical training. Due to the use of MRI scanning in this study, current pregnancy or the presence of magnetic metal in the body were additional exclusion criteria.

The original power analysis was designed to identify a difference between return-to-work proportions from 80% to 60%, and showed that n = 100 in each arm would give a power of 84%. However due to a subsequent recognition that pain and pain-related activity might be earlier and more sensitive indicators of changed behaviour resulting in earlier return-to-work, these were raised to the status of primary variables.

### Procedure

Recruitment and initial physical examinations were conducted in the period from June 2004 to October 2005. Consecutively referred patients, classified as suitable on the basis of screening criteria, were informed about the study. People were informed that the purpose of the study was to compare two different treatments for chronic/recurrent LBP: one with a primary concentration on fear-reducing information, and the other on symptom-based physical training. They were also informed that the current waiting period for assessment and treatment at the clinic was more than 3 months, whereas participation in the study would result in an MRI scan, with earlier diagnosis and treatment.

All the people who then expressed an interest in participating in the study underwent a comprehensive examination by the same clinician (PHS) lasting up to 1½ hours, during which further assessment against the inclusion and exclusion criteria was performed. A diagnosis was not given to the patient at this point, and the objective findings were explained to them in a neutral way. Patients not included received the usual treatment offered at The Back Center.

The patients who were considered eligible for inclusion were then asked to provide written informed consent and subsequently to visit a secretary who managed the randomisation, using unmarked sealed envelopes, containing a note on which was randomly written either:

• Educational programme (EDUC), or

• Physical training programme (TRAIN).

During this and following procedures, the principal investigator was not present.

After randomisation, the patients completed self-report questionnaires.

They received a schedule, including a date for an MRI scan in the following week, and an appointment for the first consultation with either a medical professor (TB) for EDUC, or with a specially trained physiotherapist for TRAIN.

### Blinding

The same investigator (PHS) managed the baseline examination and controlled the follow-up forms, blinded to the treatment group. In the data, treatment groups were named X and Y until the end of analyses.

### MRI

To avoid a possible variation across the participants in level of confidence resulting from some having had an MRI and others not, all had a standard lumbar MRI (0.2 T MRI-system, Siemens Open Viva). Two experienced claustrophobia and failed to undergo the scan. One patient had a pelvic MRI only, because her symptoms were located around the sacro-iliac joints. The imaging protocol consisted of one localiser and four imaging sequences. Most patients demonstrated degenerative changes. More detailed imaging results will be published separately.

### Interventions

During the first visit, both groups received an additional specific physical examination. In the EDUC group this was short, and mostly directed towards possible tense and tender muscles and a fear-avoidance movement pattern. In the TRAIN group, possible directional preference and neuromuscular stability were tested (see below). In both groups, explanations of the MRI scan, of the objective findings from the baseline examination, and if possible, a clarification of the pathology causing the patient's symptoms, were given. Especially in the EDUC group disc degeneration was explained thoroughly, but in both groups it was emphasised that the relationship between disc degeneration and pain is weak.

#### 1. The educational programme

The educational approach was adapted from Indahl[9] and is described below. The participants attended one to three 30-60 minute sessions, at one to three week intervals, the first and third of which were carried out by TB. The second was a one-hour group session with approximately five to seven participants, often accompanied by a relative, and led by a physiotherapist experienced in chronic pain management. They were also given a CD with a PowerPoint presentation for studying at home on general biological and cognitive aspects of back pain, as described below.

Initially, the patient's perception of his/her back problem was mapped, for example, its course, the way he/she handled it, the precautions he/she was taking, and the prognosis. The goal was to give the patient a new insight, if needed, aimed at changing his/her perception towards one that was less focussed on the current LBP condition and instilled more confidence in managing the condition into the future.

Information on pathoanatomy and physiology included the view of their own lumbar MRI scan, emphasising the positive aspects rather than focusing on possible abnormalities, unless they had particular significance. They were informed that pain episodes from high-load movements are temporary and do not cause permanent damage.

It was emphasised that pain has a physical cause, whether it could be found or not. Tension could increase pain by stressing tender joints and/or by primary muscular pain. Awkward movements could also occur if they were carried out with overly conscious control, in contrast to natural movements, especially when accompanied by fear of pain. Several simple metaphors were used to help reinforce this message. Such information may assist in the performance of more natural spinal movements and accordingly result in less pain. This mental attitude requires an understanding of the above mechanisms.

One specific back-muscle stretching (seated flexion + rotation) exercise was practised with the patients.

#### 2. Symptom-based physical training programme

At the first consultation, the physiotherapist began with a complete Mechanical Diagnostic Therapy (MDT) examination to find a possible directional preference. In the case of centralisation (where radiating pain shortened its radiating distance) or just pain relief with this procedure, patients were treated with the relevant directional-preference exercises, along with advice on optimal postures. The emphasis was on gradual progression with an attempt to eventually regain full function.

**Figure 1:**
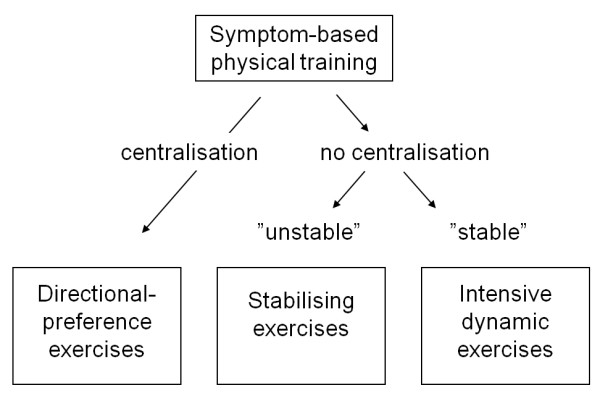
**The symptom-based flow in determining the individual treatments**.

If such MDT testing was negative (Fig. 1), the patient was tested for neuromuscular stability.

This included assessing the patient's usually painful movements when also performing so-called stabilisation procedures. If he/she could not activate the trunk muscles appropriately, or had pain relief by pretension of the trunk muscles during exercise, they were deemed 'unstable.' Accordingly, an individual programme was initiated with an emphasis on regaining stability. The exercise regime went from muscular stabilisation in basic neutral positions towards stabilisation in everyday functions.

If patients were not classified as having a directional preference or as being unstable, they were assigned to an intensive dynamic exercise programme. This programme emphasised adequate balance, endurance and strength exercises of the trunk muscles, fitness training and 'therapy-ball' exercises. This programme was conducted in a group setting but was concluded with an assessment of each individual's final muscle control.

In addition, participants in the TRAIN group were treated in a 'best practice' manner that augmented their physical training with other therapies. This meant that several health professionals could be involved as deemed relevant by the physical therapist: a nurse (medication or pain management), a chiropractor (manipulative therapy), a doctor (steroid injection) or conferences (multi-disciplinary approaches to pain management) in continued treatment plans. Each patient also received individual recommendations for further training at home, outside or in a club/training centre.

The physical training programmes were managed by two physiotherapists, one who was a credentialled MDT therapist, and the other who had attended all MDT courses but was not yet a graduate. They had completed training courses in kinetics control and had several years' experience with cLBP patients.

## Assessment and outcomes measures

The patients completed questionnaires at baseline and at 2, 6 and 12 months after the end of the treatment. Because treatment duration differed, we deemed the treatment period to end 2 months after the first examination. In other words, the 2-month follow-up period covered 2-4 months after baseline.

Baseline and/or outcomes data were collected on the following clinical characteristics:

***Demographic data ***: gender, age, marital status, education, occupation, sick leave, level of physical activity (sport, gardening etc).

### Primary outcomes

#### Pain intensity [42]

Numerical Rating Scale 0 (no pain) -10 (as bad as could be), averaged over the preceding two weeks.

#### Activity limitation ('LBP Rating Scale')[42]

a 15-item questionnaire covering activity limitation averaged over 2 weeks, each item scoring 0, 1 or 2 (significant complaints); range 0-30. These two outcome measures have had widespread use over the past two decades, especially in RCTs from Denmark.

#### Roland Morris Disability Questionnaire (RMQ)[43]

a 23-item tool, where each question is scored 0 (no disability) or 1 (some disability). The RMQ was undertaken only at baseline enabling a comparison with other studies.

### Secondary outcomes

#### LBP attitudes

***Fear-Avoidance Beliefs Questionnaire (FABQ) *** [44]: assessed the patient's beliefs about work and physical activity as influenced by back trouble. Score 0 (lowest) to 6 (highest level of fear-avoidance belief). Of two subscales, a 4-item physical activity scale (score range 0-24) was used only. The work-related subscale was not used, since not all patients were employed. Fear avoidance was also assessed with Hasenbring's method, which will be reported separately.

***Back Beliefs Questionnaire *(BBQ)**[45]: comprising 9 items reflecting beliefs about the consequences of LBP. Each item of the BBQ was scored 0 (lowest) to 6 (highest impact).

***Physical activity ***: They were asked how many minutes weekly they had spent on several listed physical activities from intensive sport, to biking/walking, to gardening etc.

***Work ability ***: an 11-item scale on the patient's work situation[46]. The answers were dichotomised afterwards into able to work or not. It is presented as 'percentage of the group capable of work'.

***Quality of life *** [46]: after treatment (2, 6 and 12 months) the patient reported if his/her quality of life was: much better, better, the same, worse or much worse as a consequence of the treatment.

***Use of medical services: *** the numbers and types of LBP-related treatments received in addition to those in the project, and during the whole study period (GP, chiropractor, physiotherapist, hospitalised/surgery or other therapist). Use of pain medication was divided into 5 groups: Nothing/weak pain killers or morphine-containing medication, both 1-4 or 5-7 days weekly.

### Outcomes to assess other treatment aspects

***Number of sessions during the study treatments***: treatment costs were estimated by counting the numbers of treatments the patients in the two treatment arms received.

***Treatment preference: *** to assess whether patient treatment preferences had an influence on LBP outcomes, patients were asked, before randomisation, which intervention they would prefer being allocated to.

### Data Analysis

Data from the questionnaires and MRI evaluation forms were entered into a database using Epidata 3.1 (Epidata Association, Odense, Denmark) using double data entry. Errors were corrected by a secretary who was blinded to treatment allocation. Data were transferred to SPSS (SPSS Base 14.0) for statistical analysis.

### Statistical analysis

Demographic variables are presented as summary statistics: medians and inter-quartile ranges (IQR) are used for continuous and ordinal variables. To facilitate inclusion in meta-analyses, outcomes are also reported as mean and SD despite their skewed distribution. Frequencies are reported for binary variables. The primary endpoints were reduction in pain and activity limitation. Treatment groups were compared using an ANCOVA analysis with adjustment for baseline values. As a sensitivity analysis of missing data for the primary and essential secondary outcomes, another ANCOVA analysis was performed, where missing values were imputed with the last observation carried forward. This could be viewed as an intention-to-treat analysis. For changes over time within each treatment group, Friedman's Test was used except for work ability, where χ^2 ^was performed. Treatment effect was estimated at each of the three follow-up stages for the primary and secondary outcomes. No interim analyses were planned nor performed in this study.

### Role of the funding source

The sponsors of this study had no role in the scientific process.

## Results

### Participant Flow/Study Sample

**Figure 2:**
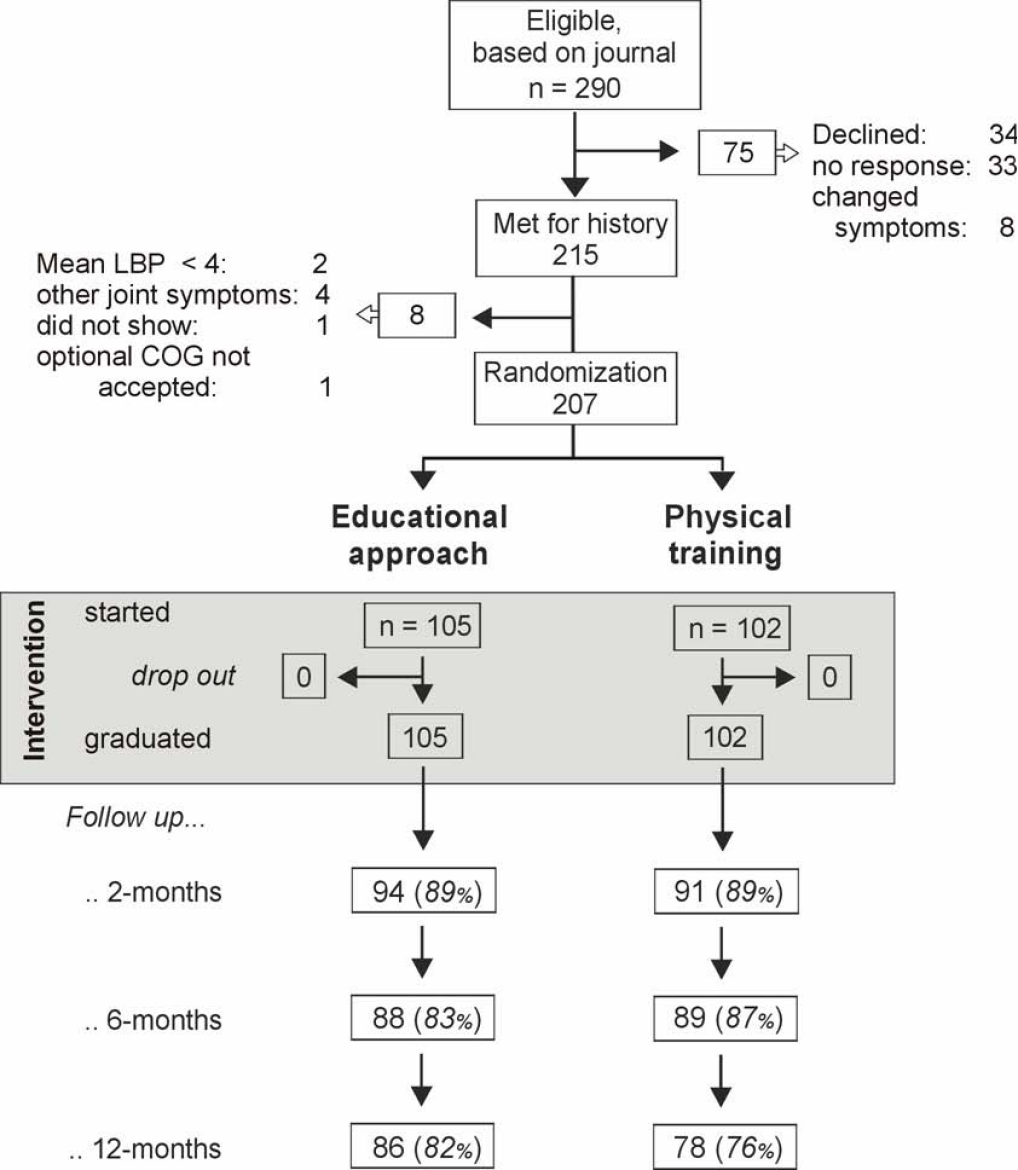
**The patients' flow throughout the study**. EDUC = educational approach.

The numbers and flow of patients are shown in Fig. 2. Participants in both groups (n = 105 and 102) were comparable at baseline, as shown in Table 1 and 2.

**Table 1:** Basic characteristics at the time of recruitment beyond the effect variables presented in Table 2

	Educational approach	Physical Training
**Age **(yrs)	40 (33-48)	38 (32-47)
**Sex **(women)	50%	55%
**BMI**	25 (24-29)	25 (23-29)
**Work ability**	70%	74%
**Rowland-Morris **(scale 0-23)	13 (9-16)	13 (10-16)
**Smokers **(yes)	35%	44%

Data are presented as medians (IQRs) or fractions

**Table 2:** Outcomes at 2, 6 and 12 months compared with baseline

	Educational approach			Physical Training			
	n	Median (IQR)	Mean (SD)	n	Median (IQR)	Mean (SD)	*p *=
Primary outcomes							
**Pain **(0(no pain) - 10)							
Baseline	103	6.1 (5-7)	6.1 (1.4)	99	6.5 (5-7)	6.3 (1.5)	
2-mths follow-up	94	4.8 (3-6)	4.6 (2.1)	91	4.7 (4-6)	4.9 (2.2)	*.7*
6-mths follow-up	87	4.3 (3-6)	4.5 (2.3)	89	5.0 (3-6)	4.8 (2.1)	*.7*
12-mths follow-up	86	4.8 (2-6)	4.5 (2.4)	78	5.1 (3-6)	4.8 (2.2)	*.8*
*Time effect*		*p <.001*			*p <.001*		

**Activity limitation (Rating Scale) **(0(best) -30)							
Baseline	104	14 (10-17)	14.0 (4.7)	98	14 (11-17)	14.1 (4.5)	
2-mths follow-up	94	11 (6-16)	11.6 (6.2)	91	13 (9-16)	13.0 (5.8)	*.09*
6-mths follow-up	87	11 (6-16)	11.2 (6.4)	87	13 (9-17)	12.7 (5.4)	*.12*
12-mths follow-up	86	11 (6-16)	11.0 (6.8)	78	13 (9-17)	13.0 (5.9)	*.09*
*Time effect*		*p <.001*			*p = .17*		

Secondary outcomes							
**FABQ (0-24)**							
Baseline	104	13 (9-18)	13.0 (6.1)	102	13 (9-18)	13.0 (6.3)	
2-mths follow-up	86	10 (6-14)	10.3 (5.9)	88	14 (9-18)	13.3 (6.4)	*<.001*
6-mths follow-up	84	11 (6-15)	10.8 (6.2)	86	13 (9-18)	13.3 (6.0)	*.007*
12-mths follow-up	84	8.5 (6-15)	10.5 (6.1)	76	13 (8-18)	13.1 (6.5)	*.01*
*Time effect*		*p = .05*			*p = .43*		

**BBQ (0-54)**							
Baseline	105	27 (18-35)	26.6 (10.9)	102	28 (20-33)	27.1 (10.2)	
2-mths follow-up	85	23 (14-32)	23.1 (10.6)	88	28 (17-36)	25.7 (13.0)	*.17*
6-mths follow-up	83	24 (14-33)	24.3 (12.7)	86	28 (22-38)	28.5 (11.4)	*.01*
12-mths follow-up	86	23 (14-34)	23.9 (12.2)	77	28 (20-35)	27.2 (11.8)	*.14*
*Time effect*		*p = .16*			*p = .13*		

**Misc. phys. activity (min/week)**							
Baseline	102	330 (180-570)	483 (525)	102	325 (199-548)	410 (307)	
2-mths follow-up	94	415 (180-600)	580 (1114)	91	360 (180-720)	561 (611)	*.90*
6-mths follow-up	85	330 (220-585)	546 (851)	87	500 (200-700)	545 (492)	*.42*
12-mths follow-up	86	310 (180-600)	419 (366)	78	390 (240-611)	480 (395)	*.19*
*Time effect*		*p = .67*			*p = .08*		

	Fraction			Fraction			
**Work ability (Yes/No)**							
Baseline (% with Yes)	73/104			75/102			
2-mths follow-up	67/93			71/91			*.35*
6-mths follow-up	64/88			68/89			*.57*
12-mths follow-up	63/86			60/78			*.59*
*Time effect*	*p = .97*			*p = .90*			

The p-values to the right refer to differences *in change over time *between groups, and the p-values below each variable refer to change over time within each treatment group. Medians are presented due to some skewed distributions.

The losses to follow-up were all due to non-attendance, even after a second written invitation. In Denmark, it is considered unethical to pressure participants beyond this.

## Outcome measures

### Primary outcome measures

#### Pain

was significantly reduced over time and approximately equally in the two treatment groups (Table 2).

#### Activity limitation

was significantly reduced during the course for EDUC (p <.001) but not for TRAIN (p = .17). A consistent trend (p = .09/.12/.09, Table 2) favouring EDUC was seen.

**Figure 3:**
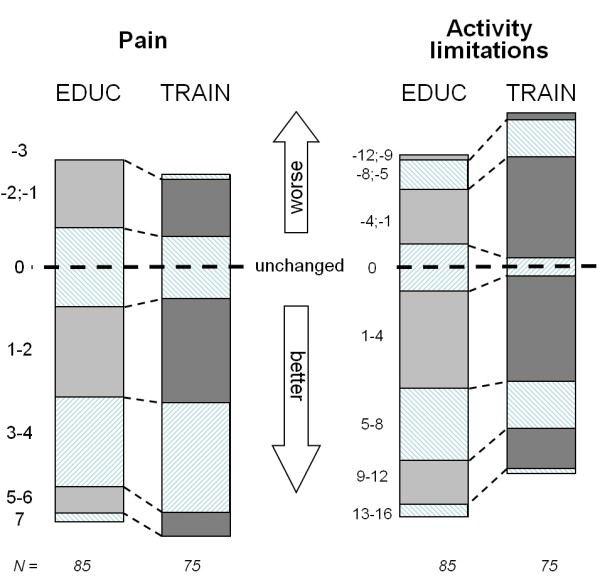
**Reduction in 'pain' and 'activity-limitation' scores from baseline to 12 months, presented by group**. E.g. (bottom left): 6 EDUC-treated patients (= grey column, numbers given by height as % of total height = 85 patients) obtained a pain reduction of either 5 or 6 compared with 5 of those in the TRAIN group. A reduction of 7 was only obtained by 2 EDUC-treated patients (lowest left area). Upper part = neg. improvement = more pain.

The distribution of the varying levels of changes in pain and activity limitation at one year is depicted in Fig. 3.

A post-hoc analysis (χ^2^) considering clinically relevant improvements (≥ 5)/"no-change" (4;-4)/worsening (≤ -5) favoured EDUC significantly at 2 months (p = .03), but not significantly at 6 and 12 months). However, a relevant question is whether there was an association between reporting less activity limitation and reduced fear-avoidance belief over time. We therefore made a post-hoc regression analysis on Δ'activity limitation' and ΔFABQ, finding significant associations at all three follow ups (p = .04/<.001/<.001), irrespective of type of treatment (p = .16/.17/.12).

### Secondary outcome measures

#### FABQ

showed statistically significant differences between the two groups at all follow-up periods, favouring EDUC. The within-group improvements over time were statistically marginally significant (p = .05) for EDUC only (Table 2). BBQ was significantly better (p = .01) for EDUC at 6 months only, but no significant time trend was observed.

Neither **reported physical activity and work ability **(Table 2) nor **quality of life, sick leave **and **medication **showed any significant differences across time or between groups. Data for the latter three are not presented.

Sensitivity analysis for the two primary measures, the two LBP-attitude measures (FABQ and BBQ) and 'misc phys. activity' revealed nothing that changed the above conclusions (data not shown).

#### Health-care contacts outside the project

There was no statistically significant difference (p = .65-.87) between the two groups regarding the total number of health-care contacts outside the project (Table 3). The slight difference in mean visits between groups at 2 months was attributed to two EDUC-treated patients having an unusually high number of visits (79 and 89 over the whole follow-up year) to other health professionals.

**Table 3:** Treatments besides those involved in the project

	Educational approach		Physical training		
**End of treatment →** **2 months**	**Numbers of visits/** **patients (responders)**	**Mean**	**Numbers of visits/** **patients (responders)**	**Mean**	**p =**

GP	95/29 (93)	1.0	92/30 (91)	1.0	
Specialist	15/4 (93)	.2	3/1 (91)	.0	
Physiother.	90/18 (93)	1.0	46/11 (91)	.5	
Chiropractor	113*/14 (93)	1.2	50/13 (91)	.6	
Others	55/12 (93)	.6	21/8 (91)	.2	
Total	368/41 (93)	4.0	212/45 (91)	2.3	.87

**2 → 6 months**					

GP	54/22 (88)	1.6	86/30 (89)	1.9	
Specialist	5/2 (88)	.3	7/3 (89)	.3	
Physiother.	99/13 (88)	3.5	93/10 (89)	3.4	
Chiropractor	61/11 (88)	2.4	49/15 (89)	1.6	
Others	74/13 (88)	2.9	65/9 (89)	2.4	
Total	276/41 (88)	3.1	271/45 (89)	3.1	.65

**6 → 12 months**					

GP	95/25 (86)	1.1	140/29 (77)	1.8	
Specialist	12/6 (86)	.1	17/10 (77)	.2	
Physiother.	103/18 (86)	1.2	132/15 (77)	1.7	
Chiropractor	139/15 (86)	1.6	59/10 (77)	.8	
Others	90/15 (86)	1.1	51/6 (77)	.7	
Total	439/45 (86)	5.1	401/46 (77)	5.2	.83

The total number of visits are presented as related to those patients making these visits, as well as medians (and IQRs) and means across all those who answered. * One patient had 50 treatments.

### Outcomes of other treatment aspects

#### Number of treatments in the project

EDUC: these patients had 1-6 sessions (median = 3, IQR = 2-3, mean = 3), each lasting between 30 and 60 minutes. TRAIN: these patients had more sessions (range = 1-20, median = 6, IQR = 4-10, mean = 7), each lasting between 30 and 60 minutes. Most treatments were the described training sessions. Forty patients had 1-2 of their treatments as a part of the multidisciplinary model, visiting a nurse for medication counselling (n = 22), seeing a chiropractor (n = 18), and/or a doctor (n = 9). Moreover, on top of the mentioned number of treatments, 22 of the patients in TRAIN compared with 0 in EDUC were discussed in multi-disciplinary conferences and 36% of the patients in TRAIN had between 1 and 6 phone consultations compared with 2% with EDUC.

#### Type of Physical Training

initially, 28% had directional-preference exercises (MDT), 42% stabilising exercises, 25% dynamic exercises, and 5% were unknown. Further treatments: 10 patients: MDT→dynamic; 24 Stability→Dynamic; 1 stability→MDT; 8 MDT→stability.

#### Patient Preference

before randomisation, 4% stated that they would prefer EDUC, and 21% preferred TRAIN; 73% had no preference while 2% didn't respond. The groups were too small for meaningful statistical analyses (Table 4), but at least fulfilling treatment preferences did not lead to better outcomes.

**Table 4:** Significance of treatment preference before randomisation on LBP and activity limitation

They preferred.. They got ....	.. educational .. educational		.. training .. educational		.. educational .. training		.. training .. training	
**LBP**	n/median (IQR)		n/median (IQR)		n/median (IQR)		n/median (IQR)	

Baseline	4	6.5 (6-7)	23	6 (5-7)	4(1)	7.5 (7-9)	19(1)	6 (5-7)
2 months	3(1)	3 (2-6)	22(1)	3 (2-6)	5	4 (3-7)	18(2)	5 (2-6)
6 -	3(1)	5 (3-6)	18(5)	4 (3-4)	5	6 (4-7)	18(2)	5 (3-6)
12 -.	3(1)	6 (3-7)	19(4)	4 (1-6)	4(1)	6.5 (6-8.5)	16(4)	5 (3-5)

**Activity limitation**								

Baseline	4	17 (15-19)	23	13 (10-15)	5	14 (6-19)	20	13 (11-16)
2 months	3(1)	13 (12-16)	22(1)	7.5 (6-15)	5	13 (9-18)	19(1)	12 (6-16)
6 -	3(1)	16 (11-16)	18(5)	10 (6-12)	5	10 (8-18)	18(2)	14 (7-16)
12 -	3(1)	16(15-18)	19(4)	10 (3-12)	4(1)	12 (11-20)	16(4)	11 (9-17)

For those 52 having indicated a treatment preference before randomisation, the numbers in each of the four possible combinations (columns) are presented to the left, with those who did not respond given in brackets where relevant. LBP and activity limitation are given as medians (and IQRs).

### Non-participants

Patients who initially didn't respond to our written project invitation or refused to participate (n = 81) were comparable with those who consented with regard to age, gender, BMI, LBP and activity limitation, but non-participants had less sick leave during the previous 12 months.

### Non-responders

Sixteen patients (eight in each group) didn't respond to all three follow-up questionnaires.

Comparisons of the baseline characteristics of non-responders and responders are shown in Table 5 for the most relevant variables. Of the other baseline characteristics, no obvious differences were seen except for a small trend towards non-responders being generally younger, men and smokers (data not shown).

**Table 5:** Non-responders' (those who did not respond at any follow-up) baseline data compared with that of responders

	Educational approach		Physical Training	
	non-responders	responders	non-responders	responders
**N **=	8	97	8	94
**LBP**	7 (5-7)	6 (5-7)	6 (4-7)	7 (5-7)
**Disability**	11 (10-15)	13 (9.5-17)	14 (8,5-18.3)	13 (10-16.3)
**FABQ**	9 (7-19)	13 (9-18)	10 (6-18)	13 (10-18)
**BBQ**	22 (20-38)	27 (18-35)	20 (14-41)	28 (22-33)
**Phys. activity**	180 (120-270)	336 (200-600)	140 (23-449)	330 (240-625)
**Work ability**	5/8	68/97	3/8	72/9

Data are presented as medians (IQRs) or fractions.

### Miscellaneous

No side effects were recorded within any treatment group. Numbers needed to treat analysis was not performed due to similar effectiveness of both treatments on most variables.

## Discussion

This is the first study comparing a cognitive educational method based on a non-injury model with that of contemporary symptom-based physical training. We have demonstrated that, among patients with cLBP, the educational/cognitive intervention with few consultations was at least as effective as an individualised, multidisciplinary physical-training approach. 'At least' refers to the observed overall trend of more improvement in activity-limitation with EDUC. There was a statistically significant difference at 2 months in favour of EDUC in the proportion of people improving by a Minimal Important Change (MIC) in activity-limitation (5 or more points on the LBP Rating Scale)[47]. As post-hoc regression analysis showed a relationship between improved activity limitation and improvement in FABQ, and FABQ was more improved in the EDUC group at all three follow-ups, on balance these data appear to favour the EDUC approach.

The cognitive educational approach was based on a non-injury model and had the focus on giving the patients an understanding of the robustness of the spine and the unlikelihood that any normal or even strenuous activity should cause any harm. With this understanding, unconscious neuro-muscular control is believed to encourage natural movements, as opposed to consciously controlled, often tense and awkward movements. Other cognitive interventions for LBP seem more or less to be based on the traditional injury model. By adding even a little caution at the end of such a cognitive session, the patient's likelihood of acquiring better coping strategies may be reduced.

Fear avoidance behaviour may be a natural consequence of the traditional medical model. On the other hand, we have all been reared in the traditional medical model and the chance of giving double messages is likely. In a non-injury model, one is devoid of such caution. Thus, cognitive intervention in a non-injury model poses challenges which are different from interventions based on a traditional model, and comparisons are therefore not useful.

The more marked effect of the cognitive educational approach in the Norwegian studies,[6,8,9] may be explained by a more effective handling of the cognitive components in those studies. An additional explanation is that in the early nineties, the alternative treatment had "be-careful" and "pay-attention-to-the-back" as core elements, which may have increased inappropriate pain-focussing.

Would it be more effective if physical treatment were added to the cognitive intervention? In the authors' opinion, probably not. The basic idea with the present cognitive model is to reduce undue focus on the pain. For many people, a demand for physical training increases pain-focus, especially if they are unable to follow their plan, or if they don't like physical exercise. Given that physical training in cLBP patients generally results in effect sizes of marginal clinical importance,[48,49] it is very likely that any improvement is outweighed by a potential harm to many patients' ability to cope. So far, only a few studies address this issue: Smeets et al.[50] did not find any additional effect of combined cognitive and physical treatment compared with groups where only one of these treatments was employed. On the other hand, Linton et al.[51] found a small additional effect for combined treatment. In Liddle et al.'s review on 'advice to stay active', a combination of exercises was recommended, but they did not specifically address the importance of reducing caution and pain-focus[52]. Klabert Moffett et al[53] found, when comparing a combined confidence-directed physical exercise programme added to cognitive components for fear avoiders, that such treatment was superior to general practitioner (GP) management of patients with LBP from 6 weeks to 6 months on the outcome of reduced disability. However, it cannot be concluded from that study if the physical components helped this particular subgroup, because the usually short GP visits are probably not sufficient to change attitude in a fear avoider, as compared with the 1 hour × 8 sessions in the Back-to-Fitness programme[53]. Also, their threshold of 2 as a minimally clinically important difference for the Rowland-Morris Disability Scale should conservatively have been 5[54]. However, it is possible that by adding "the exposure in vivo approach" as proposed by Vlayen would have reinforced our message and might have been particularly effective in such a non-injury model approach [55].

The physical training part of this study was chosen pragmatically on the background of what has become usual practice at a university clinic with physiotherapists who are well trained and well acquainted with evidence-based practice. The trial was not designed to prove efficiency in any subgroups, but to merely reflect current best practice at our clinic and probably also several similar clinics.

Recent studies have further investigated the components used in our study within the symptom-regulated programme[48,56,57]. The efficacy of directional-preference exercises for those who can centralise their pain has gained additional support[58,59]. Stabilising exercises have been further tested by one larger study showing an effect,[60] and three medium-sized studies finding no effect[61-64]. These findings have led to the effectiveness of stabilising exercises being questioned[65-67]. However, some further selection criteria have been elaborated since we started,[68] and therefore it could be that more accurate selection of patients for this treatment might have given different results. Intensive exercise for cLBP has also gained some further evidence[48,57,69]. Although the general effects of training seem to only have marginal clinical effect,[13,48,57] individualised symptom-based strategies such as those used in this study have gained much support during the past few years, showing effectiveness in several studies[36,56,58].

It can be argued that physical training should be supervised for a longer time period than was used in this study, and with higher loads. In support of that argument, Hayden et al. found increased effects in studies with a total of > 20 hours training, although they also interpreted the effect sizes having marginal clinical impact[48,57].

In the current study, 18 patients in TRAIN had manipulation. Should manipulation have been added systematically? According to Chou et al,[70] it does not seem to add any effect to physical training in cLBP.

However, it might be relevant in initial physical training settings to convince participants that the back can withstand quite heavy-loaded movements, and to identify possible movements that some patients fear, often unconsciously. Bio-feedback/EMG could be used to investigate this[20,71]. In our EDUC setting, only a specific back muscle stretching exercise was practised with the patients. Studies with bio-feedback/EMG to identify such possible fear-related muscle tension might be fruitfully investigated in subsequent studies.

That participants got earlier treatment and that all had an MRI might have also caused a selection bias, but this effect would have been equally distributed in the two groups. MRI was taken to reassure patients that their examination was thorough and to exclude serious pathology.

## Conclusion

A cognitive, educational intervention for cLBP resulted in at least as good outcomes as a symptom-based physical training method despite fewer treatment sessions. The outcome of this study and several corresponding studies and reviews[72-76] indicate that physical training as the core intervention for cLBP should be reconsidered.

## List of abbreviations

ANCOVA: Analysis of co-variances; BBQ: Back believe questionnaire; cLBP: chronic low back pain; EDUC: Educational programme; FABQ: Fear-avoidance beliefs questionnaire; GP: General practitioner; IQR: Interquartile range; LBP: Low back pain; MDT: Mechanical diagnostic therapy; MRI: Magnetic resonance imaging; RMQ: Rowland-Morris disability questionnaire; SD: Standard Deviation; TRAIN: symptom-based physical training programme;

## Competing interests

The authors declare that they have no competing interests.

## Authors' contributions

PHS wrote the protocol and the manuscript together with TB. Performed most practical aspects in running the study, except where blinding prevented her participation. Performed all patient investigations, organised questionnaires, and carried out the statistical analyses, as directed by LK and TB. TB gave ideas to the project. Took an active part in writing the protocol and the manuscript together with PHS and AI. Performed the cognitive treatment with all patients. CM organised the patient flow through the Center. Took part in scientific structuring of the project, and in drafting the manuscript. LK planned and instructed PHS in performing the statistical analyses. Wrote the statistical method section, and adjusted the results chapter to convey the data analyses. DL structured and supervised the symptom-based physical treatment. AI created the specific cognitive method, and trained TB and PHS in the practical aspects of running it with the patients. Took part in the writing process. All have read and approved the final manuscript.

## Pre-publication history

The pre-publication history for this paper can be accessed here:

http://www.biomedcentral.com/1471-2474/11/212/prepub
